# Brownian fluctuations and hydrodynamics of a microhelix near a solid wall

**DOI:** 10.1038/s41598-020-61451-y

**Published:** 2020-03-12

**Authors:** Silvio Bianchi, Viridiana Carmona Sosa, Gaszton Vizsnyiczai, Roberto Di Leonardo

**Affiliations:** 1 NANOTEC-CNR, Institute of Nanotechnology, Soft and Living Matter Laboratory, Roma, I-00185 Italy; 2 Physics Department, University of Rome “Sapienza”, Roma, I-00185 Italy; 3 Institute of Biophysics, Biological Research Centre, Szeged, 6726 Hungary

**Keywords:** Fluid dynamics, Colloids, Statistical physics

## Abstract

We combine two-photon lithography and optical tweezers to investigate the Brownian fluctuations and propeller characteristics of a microfabricated helix. From the analysis of mean squared displacements and time correlation functions we recover the components of the full mobility tensor. We find that Brownian motion displays correlations between angular and translational fluctuations from which we can directly measure the hydrodynamic coupling coefficient that is responsible for thrust generation. By varying the distance of the microhelices from a no-slip boundary we can systematically measure the effects of a nearby wall on the resistance matrix. Our results indicate that a rotated helix moves faster when a nearby no-slip boundary is present, providing a quantitative insight on thrust enhancement in confined geometries for both synthetic and biological microswimmers.

## Introduction

Many organism such as *E. coli*^1^ or *C. crescentus*^2^ spin one or more helix-shaped flagella to propel themselves. The combination of drag anisotropy in slender bodies and a chiral shape gives rise to a net hydrodynamic thrust on helical bodies that rotate around their axis^3^. Such a biologically evolved way of propulsion has recently inspired the development of artificial helical microrobots^4^ that are being perfected for targeted drug delivery^5–7^. The characterization of the thrust force of a rotating helix has been the subject of numerous theoretical^8,9^ and experimental studies^10–12^ in the field of low Reynolds number hydrodynamics. The absence of inertial effects is a very important ingredient in the description of dynamical phenomena at the micron scale, but what is really peculiar of the microscopic world is the unavoidable presence of Brownian fluctuations. In this respect very little has been done for chiral objects: Brownian diffusion of colloidal particles with regular shapes such as spheres, ellipsoids or rods, has been largely studied^13–18^. The study of more complex shapes has been limited to close packed clusters of spherical colloids^19–22^ or L-shaped quasi 2D microstructures^23^. Although roto-translational couplings may be present in non-chiral objects, they are not essential and disappear when we place the object’s origin on the center of hydrodynamic resistance^24^. On the contrary, helices are chiral objects with an intrinsic roto-traslational coupling that is responsible for the appearance of correlations in the Brownian fluctuations of rotational and translational degrees of freedom. These intrinsic correlations in chiral colloids have never been observed in experiments. The bacterium *Leptospira interrogans* is a natural colloidal helix whose Brownian motion over a solid substrate has been studied although without addressing the issue of roto-translational couplings^25,26^.

In this work we study the full Brownian dynamics of a micro-fabricated^27^ helix that is suspended in a fluid using optical tweezers. By a detailed analysis of fluctuations in translational and rotational coordinates we recover the full mobility matrix of the micro-helix including the off-diagonal terms that are responsible for roto-translational coupling. Finally, exploiting the high degree of spatial control provided by optical tweezers, we can systematically study wall effects on the roto-translational coupling. Based on these findings we conclude that a rotating helical propeller would move faster when a no-slip boundary is closer.

## Materials and Methods

Microfabrication was carried out by a custom built two-photon polymerization setup^28,29^. The micro-helices were created from SU-8 2015 photoresist (MicroChem Corp) using a 60x 1.4NA objective. Laser power and scanning speed were 3 mW and 8 *μ*m/s, respectively. After exposure, the photoresist sample was baked at 100 °C for 7 min, then developed by its standard developer solvent, followed by rinsing in a 1:1 mixture of water and ethanol, and finally dried with a gentle blow of nitrogen. Strong adhesion of the structures to the carrier cover glass was ensured by a layer of OmniCoat adhesion promoter (MicroChem Corp). To minimize deformations of the micro-helices due to the fluid flows and surface tension forces present during development, a protective structure composed of three walls is fabricated for each micro-helix. After development, the micro-helices are first immersed in deionized water and then detached from the glass substrate using a pulled glass pipette controlled by a micromanipulator. Free-floating helices could then be trapped by optical traps.

**Figure 1:**
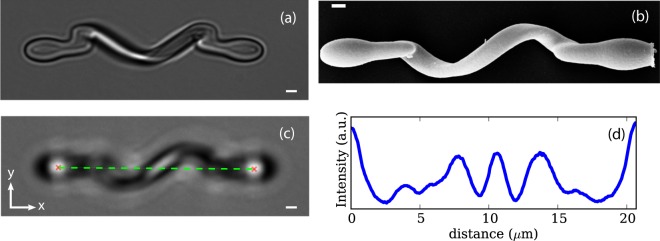
(**a**) Bright-field microscopy image of a micro-fabricated helix. At each extremity, the helix is connected to an ellipsoid with an L-shaped joint. The two ellipsoids serve as handles for two optical traps. (**b**) Scanning electron microscopy image of a micro-helix. (**c**) During the measurements optical traps are shifted 1 *μ*m above the focal plane to facilitate tracking. (**d**) A slice of the intensity of the frame shown (**b**) interpolated along the green dashed line connecting the two intensity peaks produced by the handles. The central peak, which is due to the helix, shifts as the helix rotates. All scale bars are 1 *μ*m.

A bright-field image and a scanning electron microscopy image of a micro-helix are shown respectively in Fig. 1(a) and Fig. 1(b). The micro-structure is designed as an helix with radius 2 *μ*m, pitch angle *ψ* = 42° and length $L=2\pi r\tan (\psi )=11.5\ \mu$m. At each of the two extremes, a joint connects the helix to a prolate ellipsoid with major an minor diameters of 4 and 2 microns respectively. The total length of the structure including the helix, the L-shaped joints, and the ellipsoidal handles is approximately 24 *μ*m. The micro-helices are fabricated vertically (i.e. standing up on the photoresist coated coverglass) with one of the two ellipsoid adhering on the glass. The point spread function of the writing laser is elongated along the vertical axis resulting in a helical ribbon shape (see Fig. 1(b)). A section of the helix filament can be thought as an ellipse with minor and major axis measuring 0.6 *μ*m and 1.1 *μ*m respectively.

Brownian fluctuations are observed with a standard optical microscope coupled with an IR holographic optical tweezers setup. The two ellipsoids at the extremities of the micro-helix serve as two handles that facilitate trapping by providing a strong axial gradient force that balances the scattering force on the helix filament and allows stable 3D trapping. To facilitate tracking the helices are arranged horizontally at about 1 *μ*m above the focal plane as shown in Fig. 1(c). In this configuration, the two ellipsoids produce bright blobs which can be easily tracked using a center of mass algorithm. Calling (*x*_1_, *y*_1_) and (*x*_2_, *y*_2_) the in-plane coordinates of the two handles, with *x* and *y* directions defined in Fig. 1(c), we define the helix position (*x*, *y*) as the middle point of the segment joining the two handles (dashed line in Fig. 1(c)). The in-plane orientation of the helix is computed as the angle between the line connecting the two handles and the *x* axis: $\theta =\arctan \left[({y}_{2}-{y}_{1})/({x}_{2}-{x}_{1})\right]$. The helix appears as a dark stripe with a sinusoidal shape and has a bright elongated blob given by the part of the helix that is closer to the focal plane. The rotations of the helix around its axis are measured by tracking the position of the blob respect to the handles. For each frame we interpolate the image along a line connecting the center of the two ellipsoids. Fig. 1(d) shows the image interpolated intensity along the green dashed line shown in Fig. 1(c), the peak corresponding due to the helix filament shifts upon helix rotation but is not affected by translations of the entire structure. After tracking the peak position (again using a center of mass algorithm) we convert its linear displacement Δ*l* to an angle shift Δ*ϕ* = 2*π*Δ*l*/*L*. The mean vertical distance of the helix from the coverglass can be indirectly derived as the sum of the holographic displacement of the trapping spots (1 *μ*m) and the vertical displacement of the focal plane from the coverglass (7 *μ*m). This last value can be measured by focusing on small scatterers attached to the coverslip surface and then applying a controlled *z* translation using a piezoelectric stage.

## Results and Discussion

The micro-structure is trapped as shown in Fig. 1(c) i.e. with the L-shaped arm pointing upwards. This appears to be the most favorable position in terms of tracking and stability. The trapped helix is imaged at 100 fps for almost 5 hours. We track the position and orientation of the helix through the four coordinates *x*, *y*, *θ*, *ϕ* defined above. For small fluctuations around the equilibrium configuration the force field produced by the optical traps can be derived from a quadratic potential:

1 $$U={{\boldsymbol{\xi }}}^{T}{\bf{K}}{\boldsymbol{\xi }}$$

where ***ξ*** represents the column vector (*x*, *y*, *ϕ*, *θ*)^*T*^. This last assumption is confirmed by the fact the probability distribution of all coordinates is Gaussian as expected using Boltzmann statistics. The elastic constants *K*_*ij*_ of the potential can be characterized using the well known properties of multivariate Gaussian distribution:

2 $$\langle {\boldsymbol{\xi }}{{\boldsymbol{\xi }}}^{T}\rangle ={k}_{B}T{{\bf{K}}}^{-1}$$

where *k*_*B*_ is the Boltzmann constant, *T* is absolute temperature. Replacing the average operator ⟨⋅⟩ with a time average we compute ⟨***ξξ***^*T*^⟩ from our tracks and thus obtain **K**. Indicating with **M** the mobility matrix and with **η**(*t*) the zero average white noise term we can write a Langevin equation for the helix:

3 $$\dot{{\boldsymbol{\xi }}}=-{\bf{M}}{\bf{K}}{\boldsymbol{\xi }}(t)+{\bf{M}}{\boldsymbol{\eta }}(t)$$

whose formal solution can be written as^30^:

4 $${\boldsymbol{\xi }}(t)={\bf{G}}(t){\boldsymbol{\xi }}(0)+{\int }_{0}^{t}{\bf{G}}(t-{t}^{{\prime} }){\bf{M}}{\boldsymbol{\eta }}({t}^{{\prime} })d{t}^{{\prime} }$$

where ${\bf{G}}(t)=\exp (-{\bf{M}}{\rm{K}}t)$. The time correlation function can be obtained by multiplying equation (4) by ***ξ***^*T*^(0) and by taking the ensemble average. Noting that the noise term has no correlation with the initial condition (i.e. ⟨**η**(*t*)***ξ***^*T*^(0)⟩ = 0) and using Eq. (2) to replace the term ⟨***ξ***(0)***ξ***^*T*^(0)⟩ we have that:

5 $$\langle {\boldsymbol{\xi }}(t){{\boldsymbol{\xi }}}^{T}(0)\rangle ={\bf{G}}(t){{\bf{K}}}^{-1}{k}_{B}T$$

An explicit expression of the matrix exponential **G** is complicated but its numerical computation is straightforward. Calling **Λ** the diagonal matrix storing the eigenvalues of **M**K and indicating with **C** the coordinates transform that diagonalize **M**K we have:

6 $${\bf{G}}(t)={\bf{C}}\exp (-{\boldsymbol{\Lambda }}t){{\bf{C}}}^{-1}$$

**Figure 2:**
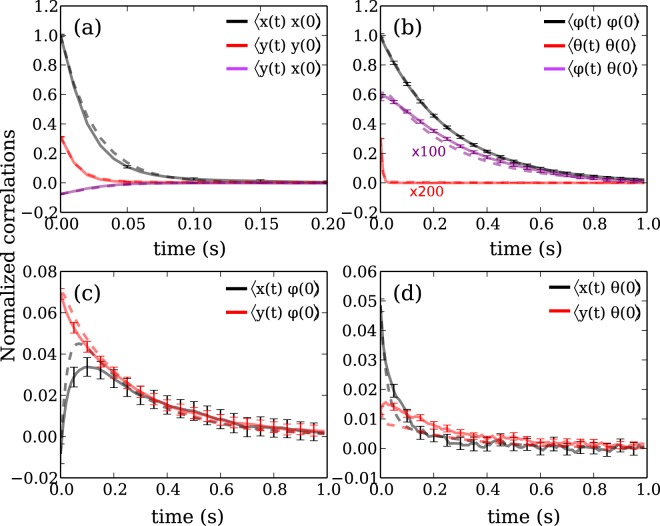
Brownian fluctuations of an optically trapped microhelix. The correlation functions have been normalized to ⟨*x*^2^⟩ in (**a**), ⟨*ϕ*^2^⟩ in (**b**), ${(\langle {\phi }^{2}\rangle \langle {x}^{2}\rangle )}^{1/2}$ in (**c**), ${(\langle {\theta }^{2}\rangle \langle {x}^{2}\rangle )}^{1/2}$ in (**d**). Purple and red curves in (**b**) have been further multiplied by factors of 100 and 200 respectively to make them visible on the same scale. Experimental data are plotted as solid lines while the dashed lines represent the best fitting curves obtained using Eq. (5).

Now that we have a way to compute the time correlations we can fit them to the data using the elements of **M** as free parameters. For each pair *ξ*_*i*_ and *ξ*_*j*_ we compute the correlation function and build the correlation matrix ⟨***ξ***(*t*)***ξ***^*T*^(0)⟩. Fig. 2 shows the measured correlation functions as solid lines. The uncertainty on each curve is calculated by dividing the trajectory into many shorter subtrajectories. For each subtrajectory we compute the correlation functions and compute their mean and the corresponding standard error (indicated by error bars in Fig. 2). The cross correlations functions are small when compared to the typical variance of each component of ***ξ*** and thus have larger uncertainties than autocorrelations.

The fitted values of **M** are reported in Table 1. The coupling term *M*_*yϕ*_ is different from zero because of the L-shaped arms connecting the helix to the handles; as the helix rotates around its axis, they L-shaped joints act as paddles pushing the fluid along the *y* axis. Such a coupling term would be zero if the tracking center coincides with the center of hydrodynamic stress^24^ but here we are considering rotations along the helix axis. Because of the symmetry of our structure, we expect *M*_*xy*_, *M*_*yθ*_, *M*_*xθ*_, *M*_*ϕθ*_ to be zero while the values reported in Table 1 are not. Such small discrepancies could originate from a minimal deformation that the helix structures may suffer when detached from the coverglass.

**Table 1:** Comparison between experimental and computed values of the mobility matrix. Experimental values have been obtained both by fitting the correlation functions (see Fig. 2) and by extrapolating the time derivative of the coordinates cross displacement for *t* → 0 (see Eq. (7)). The computed mobility matrix for our structure is obtained using a Rotne-Prager method both in the absence and in the presence of a nearby bounding wall as explained in the text. The experimental correlation functions have been obtained by averaging the correlation functions of many subtrajectories. The uncertainty on the fitted values of **M** are obtained using the bootstrap method on the subtrajectories.

Matrix term	Exp. (Correlation)	Exp. (MSD)	RP (Helix)	RP (Helix + Wall)	Units
*M*_*xx*_	10.0 ± 0.6	10.7 ± 0.3	14.2	11.5	*μ*m/(s pN)
*M*_*yy*_	6.8 ± 0.7	6.7 ± 0.2	10.5	7.7	*μ*m/(s pN)
*M*_*xy*_	0.26 ± 0.14	-0.67 ± 0.05	-0.01	0.03	*μ*m/(s pN)
*M*_*ϕϕ*_	1.6 ± 0.1	1.53 ± 0.05	1.86	1.84	(*μ*m s pN)^−1^
*M*_*θθ*_	0.094 ± 0.004	0.03 ± 0.001	0.12	0.12	(*μ*m s pN)^−1^
*M*_*ϕθ*_	0.006 ± 0.001	0.02 ± 0.001	0	0.006	(*μ*m s pN)^−1^
*M*_*xϕ*_	-0.45 ± 0.06	-0.26 ± 0.05	-0.22	-0.24	(s pN)^−1^
*M*_*yϕ*_	-0.22 ± 0.005	0.20 ± 0.02	0.36	0.3	(s pN)^−1^
*M*_*xθ*_	0.021 ± 0.001	-0.26 ± 0.015	0	0	(s pN)^−1^
*M*_*yθ*_	0.000 ± 0.001	0.40 ± 0.006	0	0	(s pN)^−1^

Since, in principle, all coordinates are coupled, each correlation function ⟨*ξ*_*i*_(*t*)*ξ*_*j*_(0)⟩ depends on all the elements of the matrix **M** so that fitting them simultaneously may be inaccurate if one of the curves presents large systematic tracking errors. As an alternative way to get **M** we can fit the cross-displacement matrix, which at short times takes the simple form:

7 $$\langle {\boldsymbol{\Delta }}{\boldsymbol{\xi }}(t){\boldsymbol{\Delta }}{{\boldsymbol{\xi }}}^{T}(t)\rangle =\langle \left[{\boldsymbol{\xi }}(t)-{\boldsymbol{\xi }}(0)\right]{\left[{\boldsymbol{\xi }}(t)-{\boldsymbol{\xi }}(0)\right]}^{T}\rangle \approx {k}_{B}T({\bf{M}}+{{\bf{M}}}^{T})t$$

The above expression tells us that, at short times, the coordinates ***ξ*** undergo a free diffusion before experiencing the effects of the quadratic potential. During free diffusion, each curve $\langle \Delta {\xi }_{i}(t)\Delta {\xi }_{j}^{T}(t)\rangle$ depends only on a single element *M*_*ij*_ of the mobility matrix. To obtain *M*_*ij*_ we numerically compute the time derivative of ⟨Δ*ξ*_*i*_(*t*)Δ*ξ*_*j*_(0)⟩. We then fit the latter to an exponential function, which empirically reproduces our data, and divide its value at *t* = 0 by 2*k*_*B*_*T*. In Table 1 are listed the elements of **M** obtained by taking the time derivative of ⟨**Δ*ξ***(*t*)**Δ*ξ***^*T*^(*t*)⟩ at *t* = *δt* (where *δt* is inverse of the framerate). Most of the values of **M** are compatible with the ones obtained by fitting the correlation functions.

**Figure 3:**
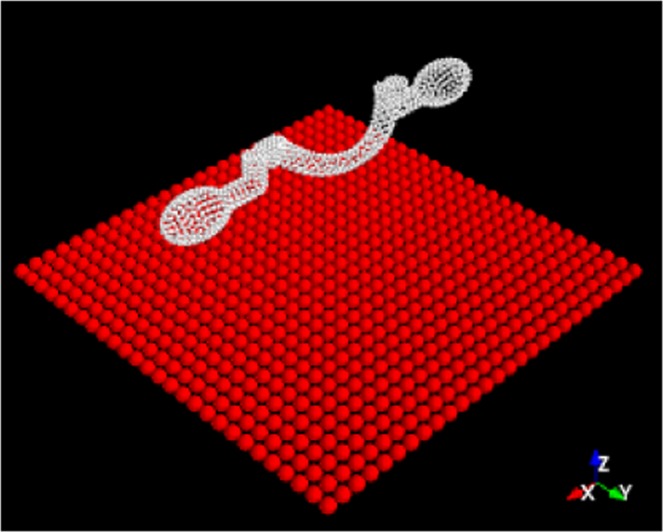
3D view of the numerical model. Both the microhelix and the coverglass surface are represented as rigid clusters of spheres. The mobility matrix of the sphere cluster composing the helical structure has been computed using the Rotne-Prager method^31,32^.

The values of **M** can be compared with the ones computed using the Rotne-Prager method. In brief the helix structure and the coverglass are represented as rigid clusters of close packed small spherical beads (see Fig. 3). For a given configuration of beads a grand mobility matrix is calculated connecting forces and torques on every bead to the linear and angular speeds of all other beads^31,32^. By imposing a rigid motion on all spheres in the cluster while setting to zero the speeds of the spheres in the wall cluster, the forces and the torques acting on each sphere are obtained by inverting the grand mobility matrix. The total force and torque on the helix are finally obtained by summing up force and torque contributions from every bead in the helix cluster. Results are listed in Table 1. When the presence of the coverglass is neglected, A significant mismatch between the measured and simulated values of the translational drag is found when the presence of the coverslip is not taken into account. The coverslip has been included in the simulation by placing a 30 *μ*m × 30 *μ*m planar layer of spheres on which we impose a zero velocity condition. In Fig. 3 we show a 3D view of the spheres composing the micro-helix and the coverslip placed at a distance of 8 *μ*m from the helix axis. Table 1 lists the values of **M** when the Rotne-Prager method also includes the coverslip effects. It is clear that the structure-wall hydrodynamic interactions play an important role. Unfortunately we were not able to increase the helix-wall distance since, as we move away from the coverslip, the optical trap strengths degrade because of spherical aberrations^33^. At approximately 10 *μ*m scattering force become dominant and the structure cannot be trapped stably for more than a few seconds.

**Figure 4:**
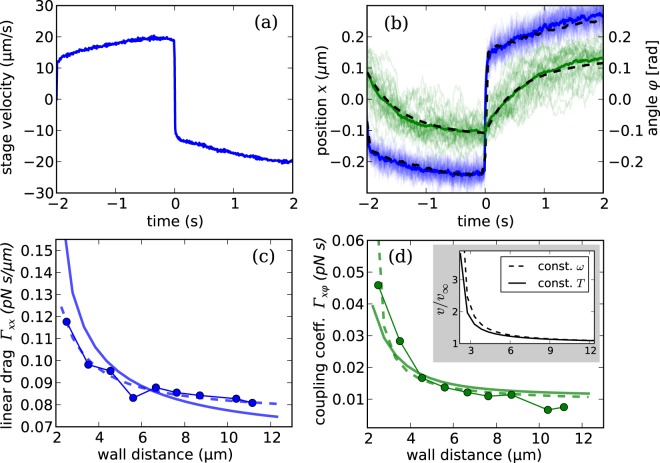
The piezoelectric stage is driven back and forth along the *x* direction while the structure is held in optical traps. (**a**) Velocity of the stage measured by its capacitive sensor. (**b**) Trajectories in *x* (blue) and *ϕ* (green). Brownian noise is filtered out by computing the average trajectory of *x* and *ϕ* plotted respectively as blue and green tick lines. Dashed lines plot a fit to the curve based on Eqs. (11) and (12). (**c**–**d**) Drag coefficients Γ_*xx*_ and Γ_*xϕ*_ as a function of the distance between the coverglass and the helix axis. The same two quantities are computed using Rotne-Prager method and plotted as solid lines. Dashed line in (**c**) plots the best fit to the function *γ*_*∞*_/(1 − (9/16)*a*/*z*) (where *a* and *γ*_*∞*_ are free parameters and *z* is the distance of the helix center from the coverslip); the same function is used to fit data point in (**d**). Inset in (**d**) plots the predicted linear velocity of an helical structure that rotates with a constant torque (solid line) or a constant speed (dashed line) at different wall separations.

Having assessed the importance of hydrodynamic effects of the wall on the structure, we now want to study the dependence of the mobility as a function of the distance. In particular we are interested in the roto-translational coupling coefficient. To do this, we trapped our structure at a controlled distance from the coverslip and tracked it while the piezoelectric stage translated the sample along the *x* axis back and forth. Figure 4(a)shows the velocity *v*_stage_ of the piezoelectric stage as a function of time. Each back and forth cycle was repeated 40 times for each distance. Figure 4(b)shows 40 tracks of *x* and *ϕ*, each corresponding to a back and forth cycle, plotted respectively as blue and green thin lines. The tracks can be averaged to eliminate the Brownian noise and obtain a mean *x*(*t*) and *ϕ*(*t*) which are plotted respectively as blue and green thick solid lines. If the Brownian noise is averaged out we can remove the stochastic term in Eq. (3) and include the effects of the translating stage by adding the external force **f** = **Γυ** where **Γ** = **M**^−1^ and **υ** = (*v*_stage_, 0, 0, 0):

8 $${\boldsymbol{\Gamma }}\dot{{\boldsymbol{\xi }}}=-{\bf{K}}{\boldsymbol{\xi }}+{\boldsymbol{\Gamma }}{\boldsymbol{\upsilon }}$$

As a first approximation we neglect all the coupling terms in **Γ** except Γ_*xϕ*_ and use the approximations ${\Gamma }_{xx}\dot{x}\gg {\Gamma }_{x\phi }\dot{\phi }$ and ${\Gamma }_{\phi \phi }\dot{\phi }\gg {\Gamma }_{x\phi }\dot{x}$ to simplify ((8)) to:

9 $${\Gamma }_{xx}\dot{x}=-{K}_{xx}x+{\Gamma }_{xx}v$$

10 $${\Gamma }_{\phi \phi }\dot{\phi }=-{K}_{\phi \phi }\phi +{\Gamma }_{x\phi }v$$

whose solutions are :

11 $$x(t)=x(0){e}^{-t/{\tau }_{x}}+\int dt{\prime} v(t{\prime} ){e}^{-(t-t{\prime} )/{\tau }_{x}}$$

12 $$\phi (t)=\phi (0){e}^{-t/{\tau }_{\phi }}+A\int dt{\prime} v(t{\prime} ){e}^{-(t-t{\prime} )/{\tau }_{\phi }}$$

where *τ*_*x*_ = Γ_*xx*_/*K*_*xx*_, *τ*_*ϕ*_ = Γ_*ϕϕ*_/*K*_*ϕϕ*_ and *A* = Γ_*xϕ*_/Γ_*ϕϕ*_. Although this simplified model provides a very good fit to the data in Fig. 4(b), the drag coefficients Γ_*xx*_, Γ_*ϕϕ*_, Γ_*xϕ*_ obtained from the best fit parameters *τ*_*x*_, *τ*_*ϕ*_ and *A* may not be quantitatively accurate because of the approximations involved. Since the trapping force field is linear in the explored range of displacements, we can follow a more straightforward way to extract Γ_*xx*_ and Γ_*xϕ*_ which does not require the above approximations on the drag coefficients. After an exponential transient the average displacements of all the coordinates ⟨*ξ*_*j*_⟩ are proportional to the stage velocity i.e. ∑_*j*_*K*_*ij*_⟨*ξ*_*j*_⟩ = Γ_*ix*_*v*_stage_. For each distance from the coverglass, a 5 minutes movie at 100 fps was acquired while the stage was not moving and *K*_*ij*_ is obtained as in Eq. (2). The values of Γ_*xx*_ and Γ_*xϕ*_ are plotted respectively as blue dots in Fig. 4(c)and green dots in Fig. 4(d). The same two quantities computed using Rotne-Prager method are also plotted in Figs. 4(c–d)as solid lines. The two curves are comparable but the agreement is not perfect. The drag on a sphere of radius *a* moving parallel to a no-slip wall at a distance *z* from the sphere’s center can be approximated by *γ*_∥_ = *γ*_*∞*_/(1 − (9∕16)*a*/*z*)^34^ where *γ*_*∞*_ is the the drag in the bulk. Using this expression as a phenomenological fitting function we can fit the experimental values of Γ_*xx*_ (dashed line in Fig. 4(c)) and obtain the effective parameters *a* = 1.75 *μ*m and *γ*_*∞*_ = 0.084 pNs/*μ*m. The value *a* is close to the helix radius (approximately 2 *μ*m). We fit Γ_*xϕ*_ using the same functional form and obtain the curve plotted by dashed line in Fig. 4(d)with parameters *a* = 4.3 *μ*m and *γ*_*∞*_ = 0.084 pNs. The length scale here is closer to the helix half-length (5.7 *μ*m).

Micro-helices are often used as propellers in both biological^3^ and synthetic^35,36^ micro-swimmers. The fact that Γ_*xϕ*_ increases as the helix approaches the wall implies that wall effects enhance the thrust force generated by a helical propeller rotating with a constant angular speed *ω*. Considering only the *x* and *ϕ* coordinates, the relation between linear speed *v* and *ω* for a force-free helix is Γ_*xx*_*v* + Γ_*xϕ*_*ω* = 0 so that *v* = −*ω*Γ_*xϕ*_/Γ_*xx*_. Although the drag coefficient Γ_*xx*_ also increases when approaching a wall, the self propulsion speed still grows as the wall distance decreases (see dashed line in inset of Fig. 4(d)). Biological propellers, such as bacterial flagella, are driven by a constant torque^37^. In this case both *ω* and *v* are derived by expressing the applied torque as *T* = Γ_*xϕ*_*v* + Γ_*ϕϕ*_*ω*. The resulting self propulsion speed for a fixed *T* is $v=-T{\Gamma }_{x\phi }/({\Gamma }_{xx}{\Gamma }_{\phi \phi }-{\Gamma }_{x\phi }^{2})\approx -T({\Gamma }_{x\phi }/{\Gamma }_{xx}{\Gamma }_{\phi \phi })$ where the drag coefficient Γ_*ϕϕ*_ also increases close to walls. Since we cannot measure directly Γ_*ϕϕ*_ we use simulations based on the Rotne-Prager method to obtain a reliable guess. We find that Γ_*xϕ*_ compensates for all these drag increments (Γ_*xx*_ and Γ_*ϕϕ*_) resulting in a self propulsion speed that increases near the wall as shown in inset of Fig. 4(d). A direct measurement of Γ_*ϕϕ*_ would require the possibility of either rotating the sample stage or applying a controlled torque on the helix, for example through radiation pressure^38^. The information on the drag coefficients coupling *x* and *ϕ* coordinate would still be the same due to the symmetry properties of the resistance and mobility matrices.

## Conclusions

Using two-photon lithography we fabricated a chiral structure composed by a micro-helix connected to two handles for optical trapping. We recorded the Brownian fluctuations of a trapped structure and extracted its drag coefficients by fitting the time correlation functions and the mean squared displacements of all coordinates. Our structure was then trapped at various distances from the coverslip and dragged through the fluid by moving the stage. From this last experiment we obtained the translational drag coefficient Γ_*xx*_ and the roto-translational coupling coefficient Γ_*xϕ*_ as a function of the distance from a no-slip boundary. Finally, we find that as the helix-wall distance is reduced Γ_*xϕ*_ grows more rapidly than Γ_*xx*_ and Γ_*xx*_Γ_*ϕϕ*_ and concluded that when a helix is rotated at a constant speed or it is subject to a fixed external torque its velocity grows as it moves closer to a no-slip wall.
